# Associations of aerobic and strength exercise with clinical laboratory test values

**DOI:** 10.1371/journal.pone.0180840

**Published:** 2017-10-23

**Authors:** Maren. S. Fragala, Caixia Bi, Michael Chaump, Harvey W. Kaufman, Martin H. Kroll

**Affiliations:** Quest Diagnostics, 3 Giralda Farms, Madison, NJ, United States of America; Leibniz-Institut fur Pflanzengenetik und Kulturpflanzenforschung Gatersleben, GERMANY

## Abstract

**Objectives:**

Physical exercise may affect levels of blood-based biomarkers. However, exercise status is seldom considered in the interpretation of laboratory results. This study reports the associations between habitual exercise participation and clinical laboratory test results.

**Methods:**

The effects of days per week of aerobic and strength exercise participation on laboratory test results for 26 biomarkers in young adults aged 18 to 34 years (n = 80,111) were evaluated using percentile distribution analyses and multivariate regression.

**Results:**

In both men and women, more days per week of either aerobic or strength exercise were significantly associated with lower levels of glucose, hemoglobin A1c, LDL cholesterol, total cholesterol, triglycerides, estimated glomerular filtration rate, globulin, and C-reactive protein, and significantly higher levels of HDL cholesterol, creatinine, iron, and percent saturation (all p < .05). Type of exercise or gender influenced the observed relationships with exercise frequency for total cholesterol, aspartate aminotransferase, gamma-glutamyl transferase, alkaline phosphatase, uric acid, bilirubin, and iron binding capacity.

**Conclusions:**

Physical exercise shifted the distribution of results into the direction suggestive of better health. Reported relationships may help clinicians and patients to better understand and interpret laboratory results in athletic populations and possibly re-evaluate interpretation of reference intervals for physically active populations.

## Introduction

Clinical laboratory tests provide information to screen, diagnose, and manage patient health and disease risk and status. While fitness and physical activity are recognized as important contributors to health and attenuating risk for cardiovascular diseases [1], information regarding the influence of aerobic and strength exercise participation on laboratory test results is sparse. Because clinical laboratory tests quantify key physiologic systems, changes in laboratory test values in terms of exercise frequency and type may offer a way to quantify improved fitness and reduction of risk of important chronic diseases, such as cardiovascular disease. In addition, results are usually interpreted in accordance with reported reference intervals that were established based on the middle 95% of a healthy population with similar characteristics [2]. However, some evidence suggests that physical exercise may affect levels of clinical laboratory tests, where results may fall outside of the reported reference intervals in highly trained athletes [3]. Characterization of the effects of exercise on clinical laboratory tests may offer a way to assess the significance of such observations.

About 21% of the adult population in the United States meet the physical activity guidelines for both aerobic physical and muscle-strengthening activity [4]. In this population, laboratory test results that fall outside of the typical reference intervals may actually be a healthy adaptation to exercise training, which might be indicated by observing a change of results toward values considered more desirable based on risk. Accordingly, prior research has suggested the need for reference intervals more reflective of the athletic population to avoid misinterpretation of results [3]. Thus, the purpose of this investigation was to explore the effects of type (aerobic and strength) and frequency of exercise participation on clinical laboratory tests in a large healthy young-adult population.

## Materials and methods

We evaluated the effects of self-reported frequency (days per week) of aerobic and strength exercise participation on circulating levels of 26 blood-based biomarkers commonly evaluated in medicine using linear regression models and percentile distribution analyses. In accord with ethical standards this study was deemed exempt by the Western Institutional Review Board. Exception status was deemed as the research was based on the analysis of existing anonymized participant data.

### Participants

The analytical sample included n = 80,111 adult (aged 18 to 34 y, mean = 30 y, SD = 3.5 y) employees or their spouses and partners. The working employees are from many different companies national wide who participated in an employee wellness program between December 2008 and April 2014. Of those reporting race and ethnicity data, the population was 57% female, 66% Caucasian, 19% Asian, 10% Hispanic, and 5% African American. The prevalence values of self- reported medical conditions are summarized in Table 1. Individuals with incomplete age, gender, or reported exercise frequency data were excluded from the analysis. Sample size per biomarker varied by marker, ranging from 27,715 to 45,725 for women and 14,384 to 34,325 for men depending which tests were included in the program for a given year.

**Table 1:** Prevalence of medical conditions (by self-report).

Condition	Number	%
Allergy	25,336	31.6
Anemia	5,659	7.1
Arthritis	1,774	2.2
Asthma	8,172	10.2
Cancer	506	0.6
Diabetes	2,044	2.6
Cardiovascular	4,135	5.2
Hypertension	4,066	5.1
Liver	184	0.2
Renal	240	0.3
Thyroid	3,205	4.0

### Biomarkers

Participants were instructed to fast for 8–12 hours prior to the blood collection. Blood specimens were analyzed for hemoglobin A1c (HbA1c), albumin:globulin ratio (A:G ratio), albumin, alkaline phosphatase (ALP), alanine aminotransferase (ALT), aspartate aminotransferase (AST), bilirubin (total), calcium, high sensitivity C-reactive protein (CRP), total cholesterol, creatinine, estimated glomerular filtration rate (eGFR), ferritin, gamma-glutamyl transferase (GGT), globulin, glucose, high density lipoprotein (HDL), cholesterol: high density lipoprotein ratio (CHOL: HDL ratio), total iron binding capacity (IBC), iron, low density lipoprotein (LDL), percent saturation (pctSat), protein (total), triglycerides, thyroid stimulating hormone (TSH) and uric acid. Hemoglobin A1c analysis was performed on the Cobas Integra 800. Both TSH and ferritin analysis were performed on the Siemens Centaur XP. C-reactive protein analysis was performed on the Dade Behring BNII. All other tests were performed on the Beckman Coulter Olympus 5800 platform. LDL analysis was performed as a direct LDL method. All reagents used were manufactured by the corresponding platform manufacturer with the exception of HDL which was performed using Roche reagents. Specimen analysis was performed by the Quest Diagnostics Lenexa Laboratory in Lenexa, Kansas. All allowable imprecision meets or does not exceed the College of American Pathologists (CAP) recommended allowable Total Error (TEa) for each assay.

### Physical activity

Physical activity was measured as self-reported days per week of aerobic and strength training exercise (0, 1, 2, 3, 4, or 5+ days per week). Data were collected by self-administered online questionnaire. Aerobic and strength exercise participation were evaluated with the following questions: In an average week how many times do you participate in Aerobic exercise? Response options were_ 0 _ 1 _2 _3 _4 _5 or more times. In an average week how many times do you participate in: Strength training exercise? Response options were_ 0 _ 1 _2 _3 _4 _5 or more times.

### Statistical analysis

Potential confounding effects of age and health status (defined as the absence of a health condition reported in Table 1) on exercise participation frequency were examined using ANOVA. The influence of aerobic and strength exercise participation on circulating levels of biomarkers were evaluated using linear regression models and percentile distribution analyses.

The effects of each type of exercise on the biomarkers were first visualized by comparing the percentile distribution among four types of activity level: 0 days/week for aerobic exercise and 0 days/week for strength exercise (A0 S0); 0 days/week for aerobic exercise and 5+ days/week for strength exercise (A0 S5); 5+ days/week for aerobic exercise and 0 days/week for strength exercise (A5 S0); and 5+ days/week for aerobic exercise and 5+ days/week for strength exercise (A5 S5). The exercise frequencies of 0 and 5+ days of activity were utilized for comparison in order to compare the most different groups of the sample.

For linear regression modeling, the data were first aggregated into the average biomarker level by all 36 possible combinations between each type of activity level (aerobic 0 to 5+ days/week combined with strength 0 to 5+ days/week). Regression was then performed on the summary data with average biomarker level as y, aerobic exercise frequency as x_1,_ and strength exercise as x_2_ for each biomarker and each gender respectively. Potential interaction effect between aerobic and strength activities were also examined by the effect of adding the interaction term (x_1_ * x_2_) into the model, and potential quadratic effect of exercise was evaluated by adding quadratic terms (x_1_^2^ and x_2_^2^) into the model.

All analysis was done in R, version 3.2.1 (The R Foundation for Statistical Computing, Auckland, New Zealand). The weighted least square regression was fitted in R using the “lm” function as follows: lm(y ∼ b_0_ + b_1_ ∙ x_1_ + b_2_ ∙ x_2_ + b_3_ ∙ x_1_ ∙ x_2_ + b_4_ ∙ x_1_^2^ + b_5_ ∙ x_1_^2^, weight = N), where b_0_ is the intercept and b_1_ to b_5_ are the coefficients for each term. The P value and standard errors of parameters for each group (gender and biomarker) were used to evaluate the significance of each coefficient. R squared, Cross validation, AIC and BIC were used to determine the best model for each biomarker.

For most biomarkers, the simple linear model is the final selected model based on the criteria mentioned considering statistical significance of each term and the limited number of different types of activity levels:

$$y=b_{0}+b_{1}∙x_{1}+b_{2}∙x_{2}+\varepsilon$$

Where y = linear terms + random errors

For some biomarkers, either aerobic or strength exercise did not show a statistically significant effect, so the model became one of below:

y = b_0_ + b_1_ ∙ x_1_ + ε (only aerobic activity showed significant effect)
y = b_0_ + b_2_ ∙ x_2_ + ε (only strength activity showed significant effect)

## Results

**Fig 1:**
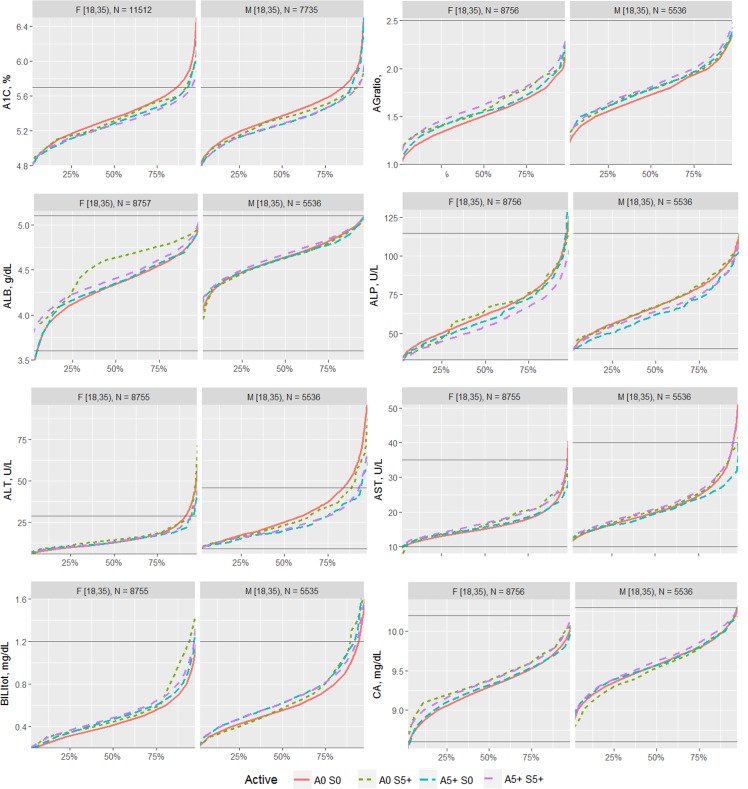
Cumulative distribution of biomarkers for 0 and 5+ days of aerobic (A) or/and strength (S) exercise participation among men and women aged 18–34 years.

**Fig 2:**
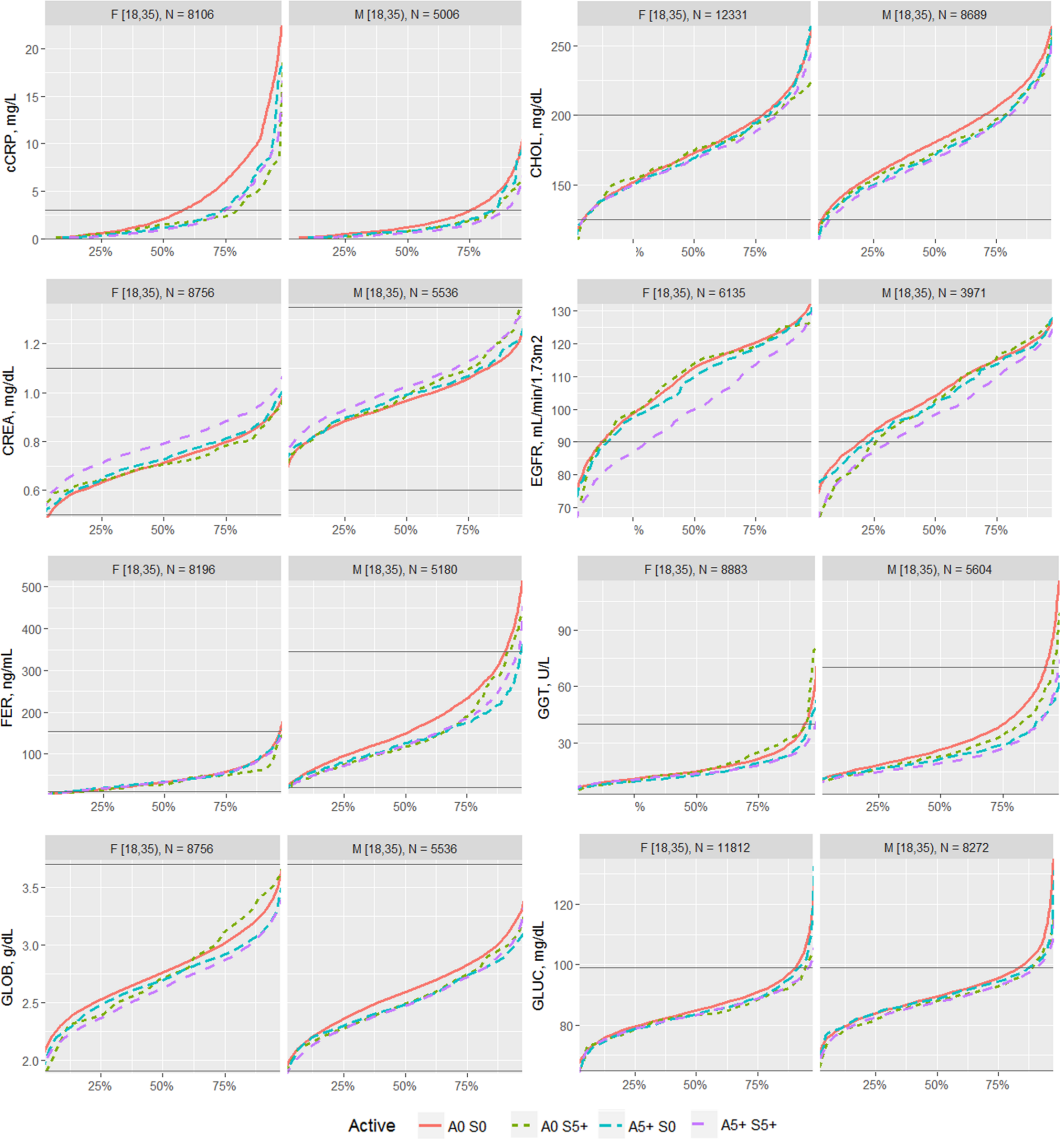
Cumulative distribution of biomarkers for 0 and 5+ days of aerobic (A) or/and strength (S) exercise participation among men and women aged 18–34 years.

**Fig 3:**
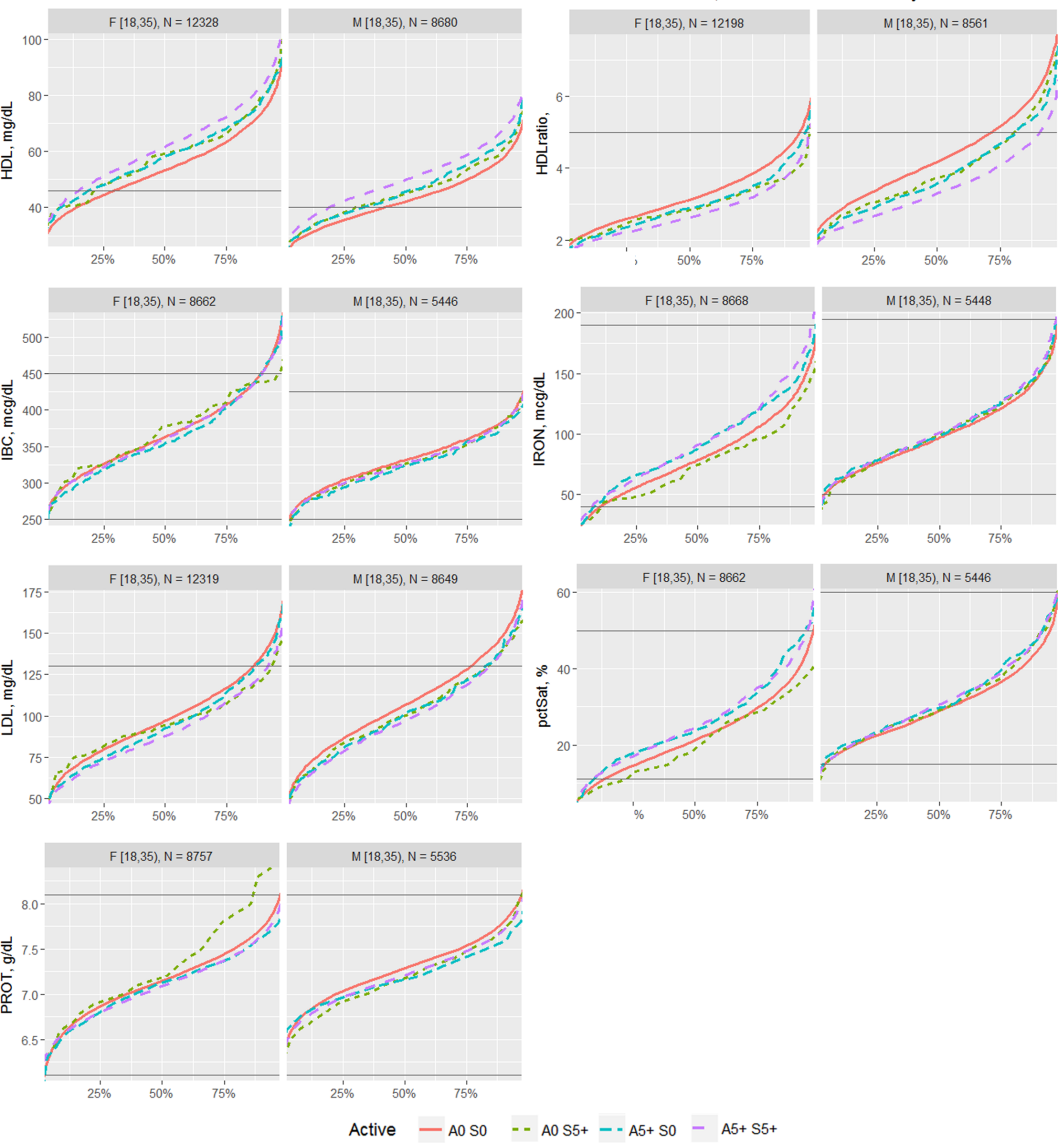
Cumulative distribution of biomarkers for 0 and 5+ days of aerobic (A) or/and strength (S) exercise participation among men and women aged 18–34 years.

**Fig 4:**
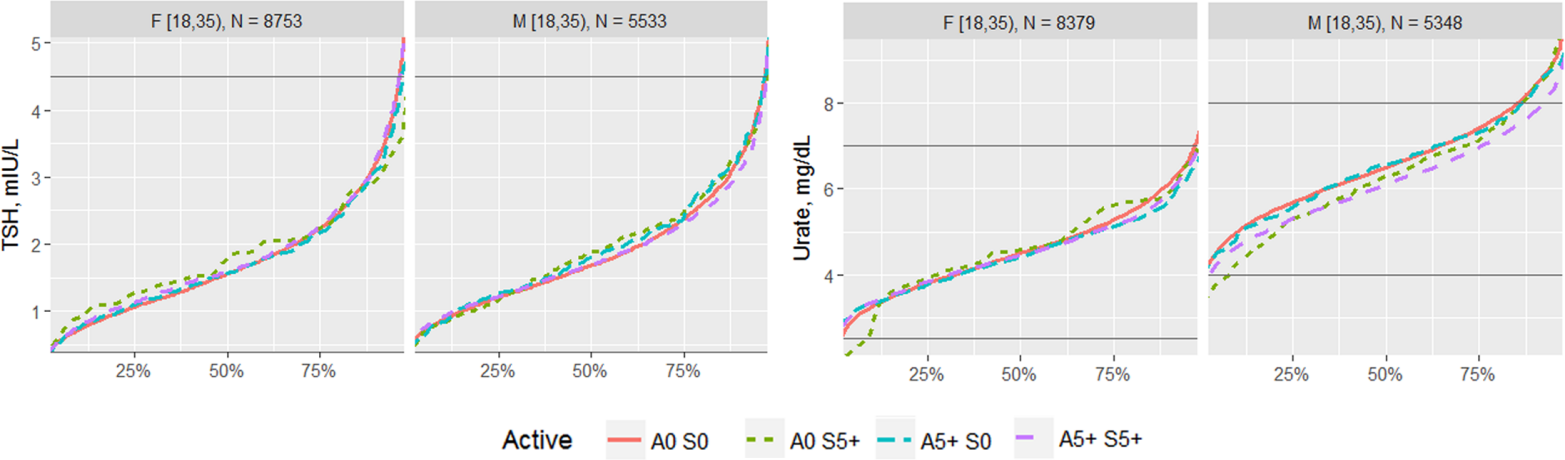
Cumulative distribution of biomarkers for 0 and 5+ days of aerobic (A) or/and strength (S) exercise participation among men and women aged 18–34 years.

**Fig 5:**
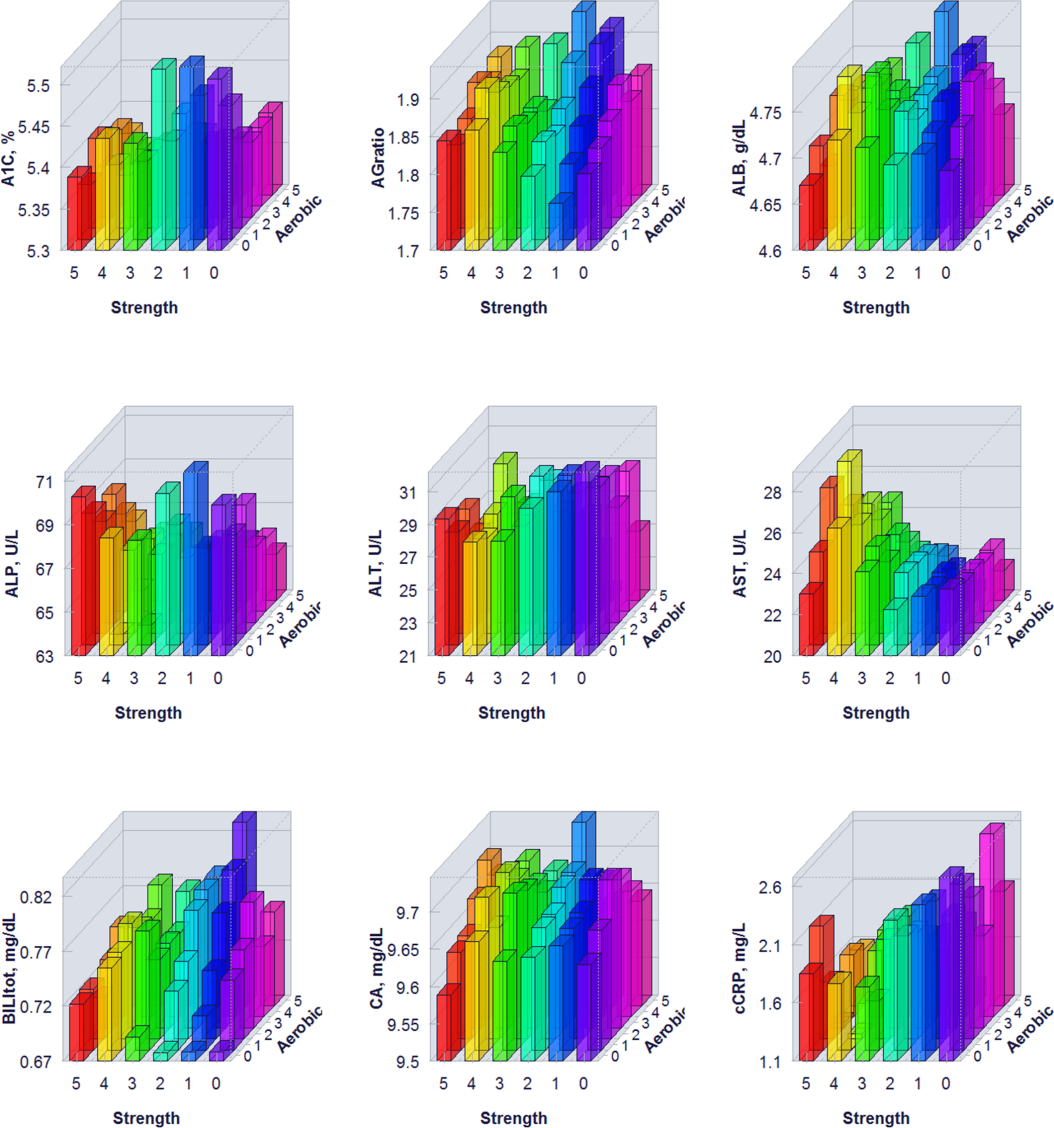
Average biomarker levels for 0 to 5+ days of aerobic or/and strength exercise participation for men.

**Fig 6:**
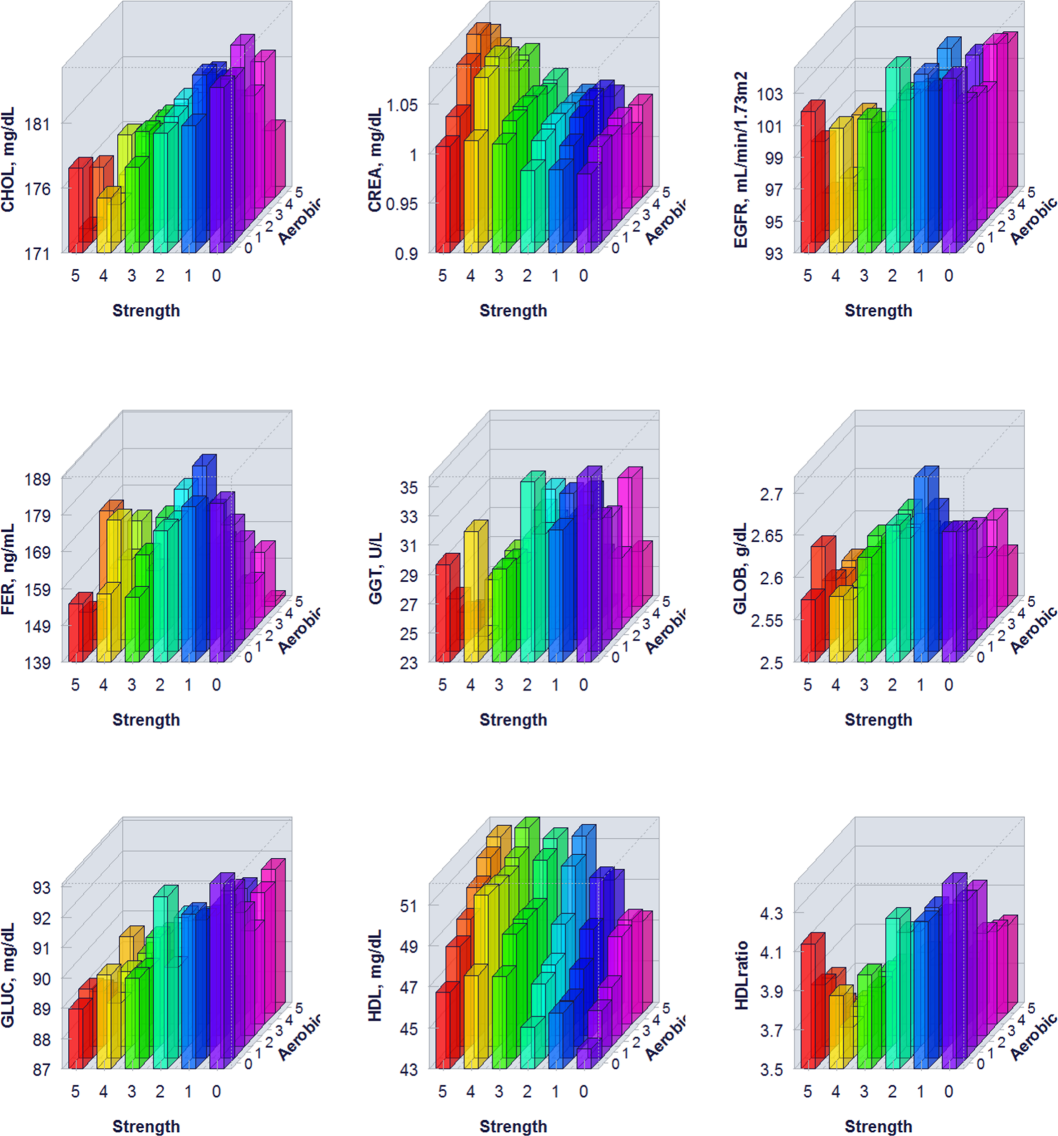
Average biomarker levels for 0 to 5+ days of aerobic or/and strength exercise participation for men.

**Fig 7:**
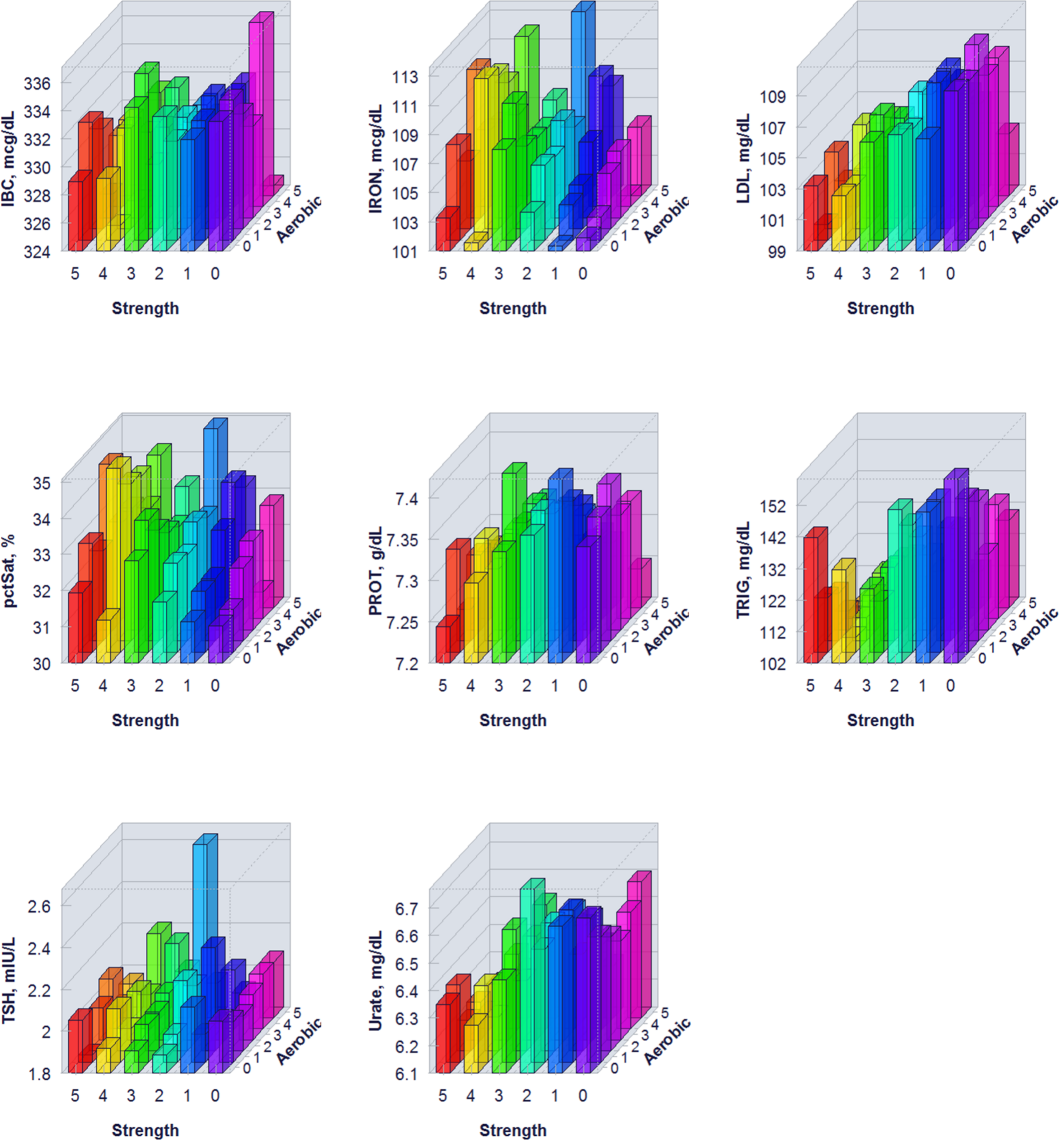
Average biomarker levels for 0 to 5+ days of aerobic or/and strength exercise participation for men.

**Fig 8:**
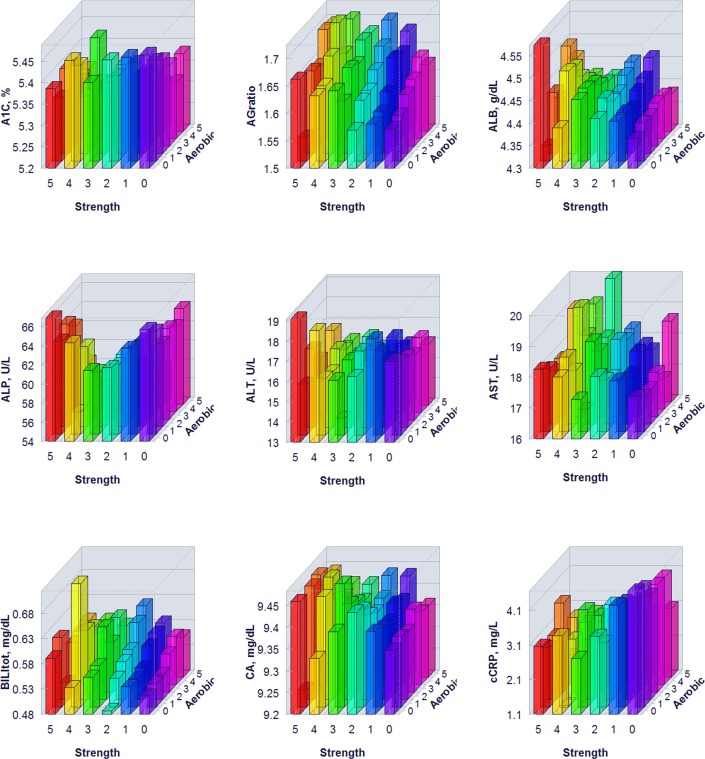
Average biomarker levels for 0 to 5+ days of aerobic or/and strength exercise participation for women.

**Fig 9:**
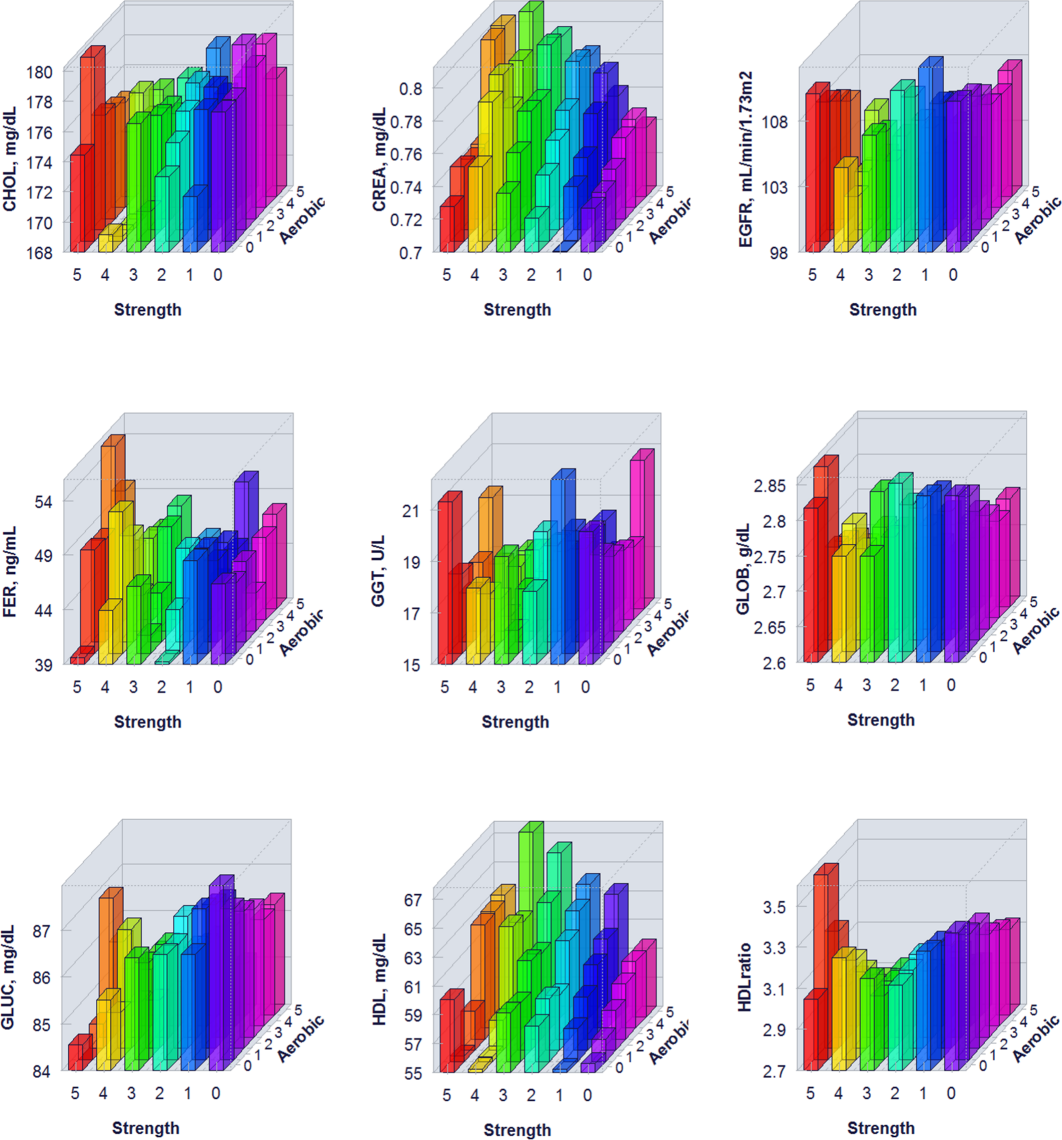
Average biomarker levels for 0 to 5+ days of aerobic or/and strength exercise participation for women.

**Fig 10:**
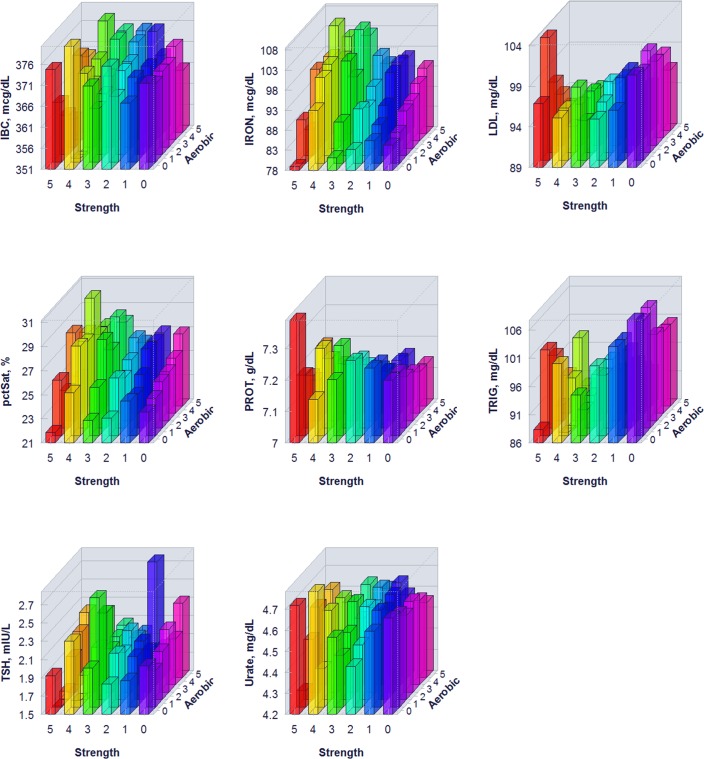
Average biomarker levels for 0 to 5+ days of aerobic or/and strength exercise participation for women.

The proportion of individuals without a reported health condition was higher for individuals reporting more days of strength or exercise participation. Younger age was associated with greater strength exercise participation, but the maximum effect in mean age was only 0.2 year for aerobic exercise and 0.67 year for strength exercise, thus clinically insignificant to account for the difference on biomarker levels. Physical exercise participation was related to clinical laboratory test results for a variety of biomarkers. The graphical presentation of the percentile distributions among the activity level groups for each marker is presented in Figs 1–4. According to the regression models (Table 2), more days of either aerobic or strength exercise were associated with lower levels of glucose, HbA1c, LDL, HDL ratio, triglycerides, eGFR, globulin, and CRP in both women and men. More days of participation in aerobic or strength exercise were associated with higher levels of HDL, creatinine, percent saturation, and iron in both men and women. Mode of exercise or gender (Figs 1–10) influenced the observed relationships between exercise frequency and cholesterol (total), bilirubin, A:G ratio, ALB, ALT, AST, ALP, calcium, ferritin, GGT, IBC, protein (total), and uric acid results. Predicted mean values based exercise frequency from the regression analysis for women and men participating in 0 and 5+ days of aerobic or strength exercise are shown in Table 3. Exercise frequency had no effect on TSH level in men or women.

**Table 2:** Multivariate regression for the effect of aerobic and strength exercise participation on biomarker levels in women and men aged 18–34 years. Sample size varied by marker, ranging from 27,715 to 45,725 for women and 14,384 to 34,325 for men. ^a^p<0.05; ^b^p<0.01 . *Italics* indicate not statistically significant.

	Women				Men			
Test	(Intercept)	Aerobic (x1)	Strength (x2)	R^2^	(Intercept)	Aerobic (x1)	Strength (x2)	R^2^
A1C	5.46	-0.0253^b^	-0.0075^b^	0.89	5.49	-0.0236^b^	-0.0082^a^	0.8
AGratio	1.57	0.0186 ^b^	0.0078^b^	0.92	1.80	0.0173^b^	*0*.*0027*	0.71
ALB	4.37	0.0062^b^	0.0133^b^	0.78	4.70	0.0086^b^	*-0*.*0026*	0.34
ALP	65.48	-1.0724^b^	-0.938^b^	0.86	69.07	-0.7420^b^	*-0*.*0563*	0.55
ALT	16.95	-0.1794^b^	*-0*.*0290*	0.37	32.05	-1.0864^b^	-0.3121^b^	0.82
AST	17.21	0.1513^b^	0.2059^b^	0.68	23.02	-0.2346^b^	0.5101^a^	0.5
BILItot	0.52	0.0122^b^	0.0075^b^	0.83	0.70	0.0167^b^	*-0*.*0014*	0.61
CA	9.35	0.0061 ^a^	0.0119^b^	0.73	9.65	0.011^b^	*-0*.*0017*	0.44
cCRP	4.60	-0.1928^b^	-0.2517^b^	0.87	2.57	-0.0957^b^	-0.1597^b^	0.8
CHOL	177.77	*0*.*2839*	-0.9137^a^	0.56	184.50	-0.5150^b^	-1.6370^b^	0.86
CREA	0.72	0.0096^b^	0.0073^b^	0.91	0.98	0.0033^a^	0.0113^b^	0.8
EGFR	110.07	-1.1619^b^	-0.8565^b^	0.9	103.67	-0.4502^a^	-0.8949^b^	0.72
FER	46.62	*-0*.*3010*	*0*.*3422*	0.05	182.75	-6.646^b^	*-1*.*9465*	0.61
GGT	19.99	-0.4522^b^	-0.2102^a^	0.71	35.15	-1.1238^b^	-1.0039^b^	0.8
GLOB	2.84	-0.0312^b^	*-0*.*0046*	0.89	2.66	-0.0206^b^	*-0*.*0059*	0.67
GLUC	87.84	-0.3543^b^	-0.3042^b^	0.88	92.66	-0.2924^b^	-0.5338^b^	0.8
HDL	55.44	1.352^b^	0.5797^b^	0.91	44.22	0.9524^b^	0.7206^b^	0.94
HDLratio	3.38	-0.0663^b^	-0.0522^b^	0.93	4.43	-0.0981^b^	-0.0926^b^	0.95
IBC	370.68	*0*.*3764*	*-0*.*5244*	0.058	333.36	-0.4295^a^	-0.5278^a^	0.48
IRON	84.08	2.602^b^	1.0756^b^	0.83	102.64	0.6744^a^	0.6371^a^	0.53
LDL	100.82	-0.6472^b^	-1.186^b^	0.85	110.03	-0.3805^a^	-1.3404^b^	0.86
pctSat	23.57	0.6684^b^	0.3202^b^	0.83	31.27	0.2454^b^	0.256^b^	0.62
PROT	7.21	-0.0222^b^	0.0087^b^	0.66	7.36	*-0*.*0065*	-0.0125^b^	0.33
TRIG	107.58	-1.8552^b^	-2.7041^b^	0.93	158.00	-6.0483^b^	-5.5925^b^	0.92
TSH	2.01	*0*.*0309*	*-0*.*0181*	0.079	2.03	*0*.*0158*	*-0*.*0183*	0.05
Urate	4.65	*-0*.*0026*	*-0*.*0098*	0.157	6.67	-0.0301^b^	-0.0523 ^b^	0.75

**Table 3:** Reference intervals and predicted values for 0 and 5+ days of aerobic or strength exercise participation per week in men and women aged 18–34 years.

		Women							Men						
Test	Unit	Reference Interval			Estimate 0 days	Aerobic 5 days	Strength 5 days	Both 5 days	Reference Interval			Estimate 0 days	Aerobic 5 days	Strength 5 days	Both 5 days
A1C	%	-	-	5.7	5.5	5.3	5.4	5.3		-	5.7	5.5	5.4	5.4	5.3
AGratio		1	-	2.5	1.6	1.7	1.6	1.7	1	-	2.5	1.8	1.9	1.8	1.9
ALB	g/dL	3.6	-	5.1	4.4	4.4	4.4	4.5	3.6	-	5.1	4.7	4.7	4.7	4.7
ALP	U/L	33	-	115	65.5	60.1	60.8	55.4	40	-	115	69.1	65.4	69.1	65.4
ALT	U/L	6	-	29	16.9	16.0	16.9	16.0	9	-	46	32.0	26.6	30.5	25.1
AST	U/L	10	-	35	17.2	18.0	18.2	19.0	10	-	40	23.0	21.8	25.6	24.4
BILItot	mg/dL	0.2	-	1.2	0.5	0.6	0.6	0.6	0.2	-	1.2	0.7	0.8	0.7	0.8
CA	mg/dL	8.6	-	10.2	9.4	9.4	9.4	9.4	8.6	-	10.3	9.6	9.7	9.6	9.7
cCRP	mg/dL	0	-	3	4.6	3.6	3.3	2.4	0	-	3	2.6	2.1	1.8	1.3
CHOL	mg/dL	125	-	200	177.8	173.2	177.8	173.2	125	-	200	184.5	181.9	176.3	173.7
CREA	mg/dL	0.5	-	1.1	0.7	0.8	0.8	0.8	0.6	-	1.35	1.0	1.0	1.0	1.1
EGFR	mL/min/ 1.73m^2^	90	-	-	110.1	104.3	105.8	100.0	90	-	-	103.7	101.4	99.2	96.9
FER	ng/mL	10	-	154	-	-	-	-	20	-	345	182.7	149.5	182.7	149.5
GGT	U/L	3	-	40	20.0	17.7	18.9	16.7	3	-	70	35.2	29.5	30.1	24.5
GLOB	g/dL	1.9	-	3.7	2.8	2.7	2.8	2.7	1.9	-	3.7	2.7	2.6	2.7	2.6
GLUC	mg/dL	65	-	99	87.8	86.1	86.3	84.5	65	-	99	92.7	91.2	90.0	88.5
HDL	mg/dL	46	-		55.4	62.2	58.3	65.1	40	-		44.2	49.0	47.8	52.6
HDLratio	ratio	-	-	5	3.4	3.0	3.1	2.8		-	5	4.4	3.9	4.0	3.5
IBC	mcg/dL	250	-	450	-	-	-	-	250	-	425	333.4	331.2	330.7	328.6
IRON	mcg/dL	40	-	190	84.1	97.1	89.5	102.5	50	-	195	102.6	106.0	105.8	109.2
LDL	mg/dL	-	-	130	100.8	97.6	94.9	91.7		-	130	110.0	108.1	103.3	101.4
pctSat	%	11	-	50	23.6	26.9	25.2	28.5	15	-	60	31.3	32.5	32.5	33.8
PROT	g/dL	6.1	-	8.1	7.2	7.1	7.3	7.1	6.1	-	8.1	7.4	7.4	7.3	7.3
TRIG	mg/dL	-	-	150	107.6	98.3	94.1	84.8		-	150	158.0	127.8	130.0	99.8
TSH	mIU/L	0.4	-	5	-	-	-	-	-		-	-	-	-	-
Urate	mg/dL	2.5	-	7	-	-	-	-	4	-	8	6.7	6.5	6.4	6.3

## Discussion

This study reports that type (aerobic and strength) and frequency of exercise are related to a variety of clinical laboratory tests in healthy young adult men and women. In many cases the direction of the influence of exercise could be suggestive of better health. Yet mode of exercise and gender influenced the relationships between exercise and biomarker results for several measures for undefined reasons that require follow-up studies. Reported relationships may help in the understanding and interpretation of common laboratory results and avoid potential misinterpretation of acceptable results that may be a healthy adaptation to exercise training. Results may contribute to the eventual generation of laboratory reference intervals that are more appropriate based on factors such as physical activity.

### Lipids (total cholesterol, LDL, HDL, CHOL: HDL ratio, triglycerides)

More days of either aerobic or strength exercise were associated with lower total cholesterol, LDL, HDL ratio and triglycerides, and higher HDL in both men and women. The association between exercise frequency and a positive blood lipid profile are in agreement with prior research [5]. Interestingly, we did not observe a significant association between aerobic exercise frequency and total cholesterol in women, despite improvements in other blood lipids. This may be due to gender differences in lipid utilization during exercise [6], a physiological threshold to support reproductive function [7], or the concurrent rise in HDL, negating a net change in cholesterol.

### Glucose & HbA1c

More days of either aerobic or strength exercise were associated with lower glucose and HbA1c in both men and women. Similarly, both physical activity and cardiorespiratory fitness have been associated with lower fasting glucose [8] and lower HbA1c [9] in prior research. Lower fasting glucose and HbA1c are indicative of better glucose regulation due to changes in beta cell functioning [8]. The association of lower glucose and HbA1c with higher exercise frequency may be due to the insulin-independent mechanism for glucose uptake by muscle cells stimulated by muscle contraction during exercise [10].

### CRP

More days of either aerobic or strength exercise were associated with lower CRP in both men and women. Being an inflammatory factor produced by the liver that increases in response to infection or inflammation [11], CRP has been associated with chronic disease and sedentary lifestyle [12,13]. Lower levels may suggest reduced systemic inflammation in more active individuals. Previous research has reported a similar association, where higher physical activity was associated with lower CRP [13]. Lower CRP is believed to be part of the mechanism for the protective effects of physical activity against cardiovascular diseases through reduced coagulation [14].

### Blood proteins (total, albumin, globulin)

Associations between exercise frequency and total blood protein differed by mode and sex. In women, days of reported aerobic exercise participation were significantly associated with lower total serum protein, while days of reported strength exercise participation were significantly associated with higher total serum protein. In men, no association between days of aerobic exercise participation and total serum protein were observed. However, days of reported strength exercise participation were significantly associated with lower total serum protein in men. Although athletes have higher dietary protein requirements to support the increased needs for protein during recovery [15], it is unclear why mode of exercise and sex appear to affect total blood proteins differently. Observations may be explained by the sub-types of blood proteins, albumin, and globulin.

More days of either aerobic or strength exercise were associated with higher levels of albumin in men and women, except for men who participated in strength training exercise, where levels were lower. Albumin is important for transporting substances in the blood such as bilirubin, calcium, and progesterone, and for maintaining osmotic balance [16]. Increased levels in active individuals may be an adaptation to exercise training to expand plasma volume [17]. Albumin is associated with protein status in the body [18], where strenuous exercise increases albumin excretion [19] and low protein intake decreases rate of albumin synthesis [20]. Men who strength train may thus have lower synthesis or higher excretion due to dietary intake [21] or reduced stimulus for albumin production.

The effects of exercise participation on blood globulin proteins showed a more consistent effect, where more days of either aerobic exercise were associated with lower levels of globulin protein in the blood for both men and women. Similarly, globulin levels have been shown previously to decrease with endurance exercise [22]. As globulin proteins consist of a variety of proteins, it is plausible that reduced globulins with exercise participation is a physiological adaptation to increase the bioavailability of different compounds in the body [23] as opposed to a reduction in immunoglobulins [24].

### Bilirubin

With the exception of strength training participation in men, more days of either aerobic or strength exercise were associated with higher levels of bilirubin that were still within normal limits. Bilirubin is a metabolite of heme produced from normal red blood cell breakdown. In healthy athletes, elevated bilirubin may indicate an accelerated rate of red blood cell turnover [25], stimulated by exercise conditions such as muscle contraction, impact, and high oxygen.

### Calcium

With the exception of strength training participation in men, more days of either aerobic or strength exercise were associated with higher levels of calcium. Prior research has shown that plasma calcium increases in response to exercise [26] likely due to metabolic acidosis [27]. Because 40% of circulating calcium is bound to albumin, the observed changes in calcium may be a direct result of the changes in albumin concentration.

### Creatinine and eGFR

More days of either aerobic or strength exercise were associated with higher levels of creatinine in both men and women. As creatinine is a metabolic product of creatine breakdown, active individuals and those with higher muscle mass would be expected to have higher creatine turnover resulting in higher serum creatinine levels. Prior research has similarly shown higher serum creatinine in athletic populations [9], particularly in sports involving strength and power [28]. As our results and prior research have shown [28], reference intervals for creatinine for the general healthy population may not be appropriate for the active population.

More days of either aerobic or strength exercise were associated with lower eGFR. eGFR is a measure of the rate that the kidneys are able to filter blood that is based on creatinine in the blood, along with age, race, and sex. While physical activity has been positively associated with kidney function [29] eGFR may be higher in athletes following exercise because of higher creatine metabolism, especially for athletes with larger muscle mass [30]. However, eGFR may be temporarily decreased in the day following exercise [31] and has been shown to be lower in some endurance athletes and team sports athletes [32].

### Iron, percent saturation, TIBC, and ferritin

More days of either aerobic or strength exercise were associated with higher levels of iron and percent saturation in both men and women. In addition to being important for the production of hemoglobin and new red blood cells, iron is an important constituent of myoglobin and various enzymes. Iron is also important to the metabolic processes involved in exercise and adaptations to exercise training [33,34]. Athletes may lose iron 20% faster than non-athletes [35], as iron red blood cells are broken down during exercise by the mechanical stress of muscle contraction [36]. Thus, the higher levels of iron and percent saturation in those with higher activity levels may be due to increased needs for iron during exercise [37].

### TIBC

The effects of exercise frequency on TIBC differed by gender. In women, neither aerobic nor strength exercise participation were significantly associated with TIBC results. However, in men, both days of reported aerobic exercise participation and days of reported strength exercise training were significantly associated with lower TIBC. TIBC is a measure of the body’s ability to transport iron in the blood and is often elevated with iron-deficiency, lower work capacity, and fatigue in athletes [38,39]. The lower levels of TIBC associated with higher levels of aerobic or strength exercise in men may be an adaptation to exercise training to allow greater iron transport to accommodate increased needs with higher exercise volumes. It is unclear why the same association was not evident in women. However, it may be due to the higher prevalence (32%) of iron deficiency [40,41] or supplementation (50%) [42] in female athletes.

### Ferritin

While days of reported aerobic exercise participation were significantly associated with lower ferritin in men only, days of reported strength exercise training were not associated with significant changes in ferritin levels in men or women. As ferritin is a protein that stores and transports iron, levels correlate to iron status in the blood. Previous research has shown that high-volume exercise training leads to decreased ferritin levels in male endurance athletes [37]. Although type and duration of exercise determine iron metabolism and blood cell adaptations [37], it is unclear why aerobic exercise were not associated with ferritin levels in women.

### Liver enzymes (ALT, AST, GGT)

With the exception of men who participate in aerobic exercise training, more days of strength or aerobic exercise were associated with higher AST. Additionally, with the exception of women who participate in strength exercise training, more days of strength or aerobic exercise were associated with lower ALT. AST and ALT are both aminotransferase enzymes found primarily in the liver and play a role in amino acid metabolism. Elevated levels of AST in active individuals are likely a result of increased amino acid metabolism and release from muscle [43]. Prior research has shown that both AST and ALT increase after both aerobic [44] and strength exercise where levels can be elevated for more than 7 days [45]. As metabolic demands are increased in active individuals, it is not known why higher frequency of participation in aerobic exercise was associated with lower AST in men or why levels of ALT tended to be lower in active individuals; these associations may be due to lower amino acid metabolism in these individuals.

More days of aerobic or strength exercise participation were associated with lower GGT in men and women. Serum GGT is derived from the liver [46], serves as an indicator of general liver health, and is transported with albumin and lipoproteins in the blood [47]. Prior research has shown that although GGT increases acutely after aerobic exercise [44], lower resting GGT is associated with higher physical activity [48]. The role of GGT in exercise may be through counteracting oxidative stress by breaking down extracellular glutathione and making its component amino acids available to cells for repair [47]. Serum levels of GGT are positively associated with body mass index, alcohol use, and total serum cholesterol [48].

### ALP

With the exception of men who strength train, more days of aerobic or strength exercise participation were associated with lower ALP in men and women. ALP is an enzyme found primarily in bone and the liver that is involved in both removal of mineral phosphate from molecules and inflammatory conditions. Levels of ALP are related to bone activity [49]. As levels of bone specific ALP increase during weight-bearing exercise [50], but return to baseline within 20-minutes following exercise, lower resting levels may be a consequence of the transient response to weight bearing exercises.

### TSH

Neither days of reported aerobic nor days of strength exercise participation were significantly associated with TSH in men or women. Thyroid hormones play an important role in metabolism, growth, tissue differentiation, fatty acid oxidation, and thermoregulation in response to exercise training [51–53]. Previous studies evaluating thyroid function in athletes have shown contradicting results [54,55]. In elite soccer players, TSH levels have been shown to increase over a competitive season [54]. However, in other studies, both power and aerobic athletes have been shown to have a lower serum TSH [55], indicating a possible increased sensitivity of the thyroid gland to TSH in athletes. Although, specific training plans may affect thyroid hormones in circulation, the present data does not support evidence of different levels of TSH in the active population compared to the more sedentary population.

### Uric acid

The effects of exercise frequency on uric acid differed by gender. In women, neither aerobic nor strength exercise participation were significantly associated with uric acid results. However, in men, reported days of both aerobic and strength exercise participation were significantly associated with lower uric acid. Lower uric acid may be an adaptation to training, as prior research has reported lower uric acid in male athletes compared to non-athletes [3]. Uric acid increases after intense exercise that recruits fast-twitch muscle fibers [56], likely in order to increase serum antioxidant capacity and reduce oxidative stress during acute physical exercise [57]. Despite its short term role in exercise responses, high resting uric acid has been associated with poor strength [58], vulnerability to tendon injury [59], and disease [60].

While this study reports new information on the relationship between exercise participation and common laboratory measures, findings should be interpreted within the context of the present study design. First, from a population level, assessment of exercise participation by frequency and mode alone is not able to capture the relationships or mechanisms with other exercise metrics such as intensity and duration. Further study is needed to evaluate the effects of exercise intensity and duration on these relationships. Additionally, in order to isolate and explore the different effects of strength vs. aerobic type exercise, predicted values were reported as 0 vs. 5+ days per week of exercise participation. While this may differ from some physical activity recommendations, it better allowed the distinct effects of each mode of exercise to be displayed. In addition, as exercise and health data were collected by questionnaire, the potential for recall and reporting bias may exist. Interpretation bias may also exist, as the questionnaire did not define aerobic or strength exercise. Furthermore, participants with reported medical conditions that may affect common laboratory tests were included in the study in order to represent a characteristic population. While the proportion of individuals without a reported health condition was higher for individuals reporting more days of strength or exercise participation, all individuals were included in the analytic sample due to broad health condition classifications, insufficient sample sizes by each reported condition to power subgroup analysis, and in order to represent a characteristic sample of the population. In regards to age, although younger age was associated with greater strength exercise participation, the restriction of our population to adults aged 18 to 34y and the less than 1 year difference found between groups was not considered clinically meaningful and supported the non-stratified analysis. Additionally, the sample may be biased toward working employees and not necessarily representative of the population. However as the goal of this study was to demonstrate the effects of exercise on biomarkers of health, the relationships may be applicable to a more broad population. Finally, this study was restricted to men and women aged 18 to 34 years of age in order to avoid the confounding influence of age. Accordingly, application of results would be most appropriate to populations with similar characteristics. Further study is needed to determine the influence of age on such relationships.

Physical exercise participation is related to clinical laboratory test results for a variety of common biomarker results. Laboratory test results should be interpreted within the context of each person and their unique set of circumstances. Reported relationships may help in the understanding and interpretation of common laboratory results for young, physically active adults and lead to defining appropriate reference intervals based on factors such as physical activity. Such data may help to interpret laboratory test results that may be a healthy adaptation to exercise training and to avoid misinterpretation of acceptable results.

## Supporting information

S1 File Data for submission_20170512.xlsx. (XLSX)

## Abbreviations

A1C: hemoglobin
A1c: (A:Gratio), albumin: globulin ratio
ALB: albumin
ALP: alkaline phosphatase
ALT: alanine aminotransferase
AST: aspartate aminotransferase
BILItotal: bilirubin, total
CA: calcium
cCRP: cardiac C-reactive protein
CHOL: cholesterol
CREA: creatinine
eGFR: estimated glomerular filtration rate
FER: Ferritin
GGT: gamma-glutamyl transferase
HDL: high density lipoprotein
HDLratio: cholesterol: high density lipoprotein ratio
IBC: iron binding capacity
LDL: low density lipoprotein
pctSat: iron percent saturation
PROT: protein
TRIG: triglycerides
TSH: thyroid stimulating hormone
